# Hydatid Cyst of Spleen Presenting with Vague Symptoms: A Diagnostic Conundrum

**DOI:** 10.7759/cureus.5815

**Published:** 2019-10-01

**Authors:** Jawad Ahmed, Taha Bin Arif, Faryal Tahir, Farheen Malik, Oam Parkash

**Affiliations:** 1 Internal Medicine, Dow University of Health Sciences, Karachi, PAK; 2 Pediatrics, Dow University of Health Sciences, Karachi, PAK

**Keywords:** splenic cyst, echinococcus, hydatid cyst, parasitic infestation

## Abstract

Hydatid cysts are caused by a tapeworm Echinococcus granulosus. They usually occur in the liver. When occurring in spleen they present with vague symptoms which make it difficult for the physicians to diagnose. We present a case of a 10-year-old male who presented with fever, abdominal pain, and burning micturition along with vomiting. Abdominal exam revealed no visceromegaly. Abdominal ultrasound (US) and computed tomography (CT) scan showed multiple hypoechoic and hypo-dense areas, respectively. Splenic abscess, abdominal tuberculosis (TB), pyelonephritis and malignancy were ruled out with appropriate investigations. The patient was not responding to triple therapy of antibiotics (ceftriaxone, metronidazole, and cloxacillin). A final diagnosis of hydatid cyst of spleen was made when serum echinococcus immunoglobulin G (IgG) antibodies were found to be positive. The patient was treated with albendazole and was discharged on improvement. It is vital to be vigilant and consider echinococcal hydatid cyst as a differential in the lesions of spleen, especially if the patient has a rural background.

## Introduction

Echinococcosis or hydatid disease is caused by the parasitic tapeworm Echinococcus granulosus in its larval form. Primarily a disease of sheep and cattle, humans are an accidental intermediate host in their cycle of development. In countries like Pakistan, in which sheep and cattle rearing are considered the livelihood of many, it is an endemic disease. Hydatid disease can be found in any organ of the body but most commonly it affects the liver (60-70%), lungs (30%), and to a very rare extent the spleen, kidney, pancreas, bone, and thyroid. Although splenic hydatidosis has been reported as early as 1790 by Berlot as an autopsy finding [1], its occurrence even in endemic areas is less than 3%. The rarity of splenic hydatid cyst, the diagnostic dilemma it poses and its isolated presence in the spleen makes this an interesting case to report.

## Case presentation

A 10-year-old male, completely vaccinated, resident of rural Sind, presented in outpatient department (OPD) at pediatric unit in Civil Hospital Karachi (CHK) with complaints of fever and left-sided abdominal pain for a month and burning micturition for 10 days. Initially, fever was high-grade (102°F-103°F), continuous, gradual in onset, and associated with a non-productive mild cough. It was not associated with rigors and chills. Over the week following the onset, fever became low grade and was relieved by antipyretics. Abdominal pain was dull, mild in severity, continuous, and non-radiating. It was not exaggerated by the intake of meals. The child then suddenly developed burning micturition associated with vomiting. There was no complaint of retention or blood in the urine. For these complaints, the child was previously admitted to another hospital where he developed urinary retention during his course of stay and was catheterized. He was given intravenous (IV) augmentin and ciprofloxacin for four days and then referred to CHK. The child was a progeny of consanguineous marriage and was developmentally appropriate for his age. There was a history of loss of appetite and undocumented weight loss. Patient’s antenatal and postnatal histories were insignificant and all siblings were normal and healthy.

On general physical examination, the child was pale but his height (134 cm) and built was appropriate for his age. His vitals were stable with a low-grade fever (100°F). The fronto-occipital circumference was 50.5 cm. Deep palpation of the abdomen showed tenderness only in the left hypochondrium but no palpable mass could be appreciated. The abdomen was normal in appearance, with no visceromegaly and gut sounds were audible. Examination of the rest of the systems was insignificant.

Splenic abscess, abdominal tuberculosis (TB), pyelonephritis and abdominal malignancy were considered as differentials in diagnosis, keeping in view the signs and symptoms of fever, left hypochondrium pain, and weight loss. Different laboratory investigations were done to reach a final diagnosis. Complete blood count (CBC) showed anemia with hemoglobin (Hb) of 9.2 gm/dL, raised total leukocyte count (TLC) of 14.5 × 10^3^/µL, and a normal platelet count of 304 × 10^3^/µL. Urinalysis was normal with no casts and culture growth. Blood culture was also negative. Erythrocyte sedimentation rate (ESR) and C-reactive protein (CRP) were raised, 79 mm/hr and 28.5 mg/L, respectively. Montoux and Genexpert done for TB turned out negative. Viral markers for hepatitis B and C were also negative. X-ray abdomen kidney ureter bladder (KUB) showed no abnormality.

**Figure 1:**
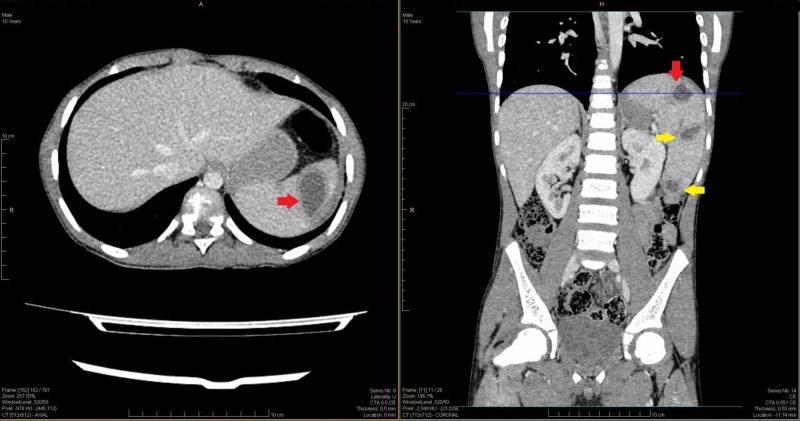
Axial and coronal view of abdominal CT scan showing multiple hypo-dense areas of hydatid cysts (red and yellow arrows), largest cyst (red arrow). CT: Computed tomography

**Figure 2:**
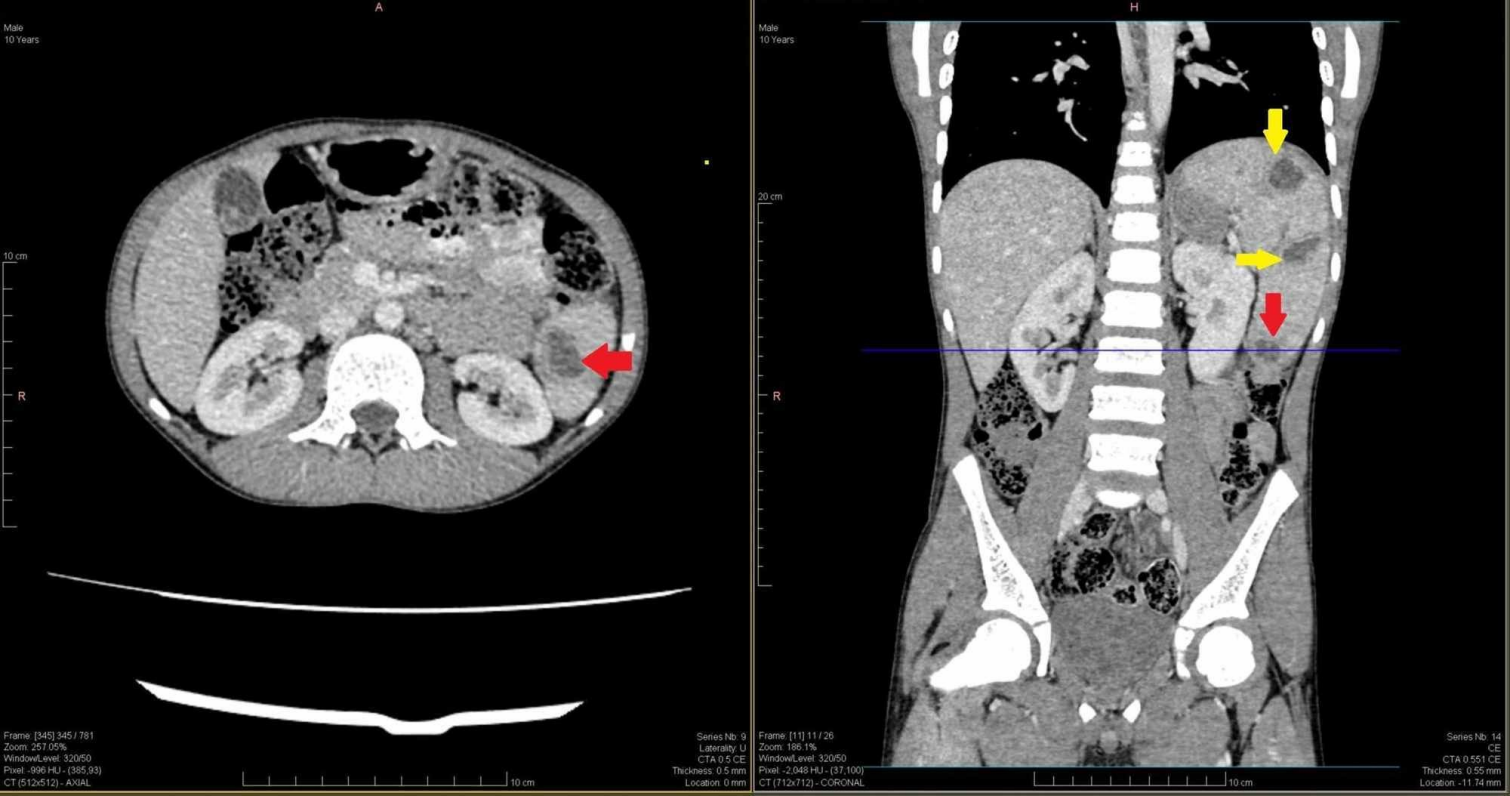
Axial and coronal views of abdominal CT scan showing multiple hypo-dense areas of hydatid cysts (red and yellow arrows), most inferior cyst (red arrow). CT: Computed tomography

The child was initially managed by intravenous (IV) ceftriaxone, omeprazole, and paracetamol. Ultrasound (US) abdomen showed a spleen of 10.7 cm with multiple hypoechoic lesions over splenic parenchyma. No vascularity was seen in the lesions on Doppler US. Lesions were suggestive of abscess, infarct or a cyst. Contrast-enhanced computed tomography (CT) scan of the abdomen revealed multiple ill-defined hypo-dense areas in the spleen with the largest one measuring 2.4 × 2.3 cm, suggestive of a splenic abscess (Figures 1, 2). Abdominal lymphadenopathy with multiple enlarged lymph nodes at para-aortic, peripancreatic, perisplenic and mesenteric region was seen. Largest lymph node at mesenteric region measured 1.8 cm short-axis diameter (SAD) and in perisplenic region measured 1.9 cm SAD. In the meantime, patient’s fever failed to subside thus IV cloxacillin with metronidazole was added with the ongoing regimen.

An opinion was sought from pediatric surgery department. They advised to continue triple regimen (ceftriaxone, metronidazole, and cloxacillin) and test for the presence of serum echinococcus antibodies. Enzyme-linked immunosorbent assay (ELISA)-based detection of immunoglobulin G (IgG) antibodies for echinococcus was positive and indicative of echinococcus infection. A final diagnosis of hydatid cyst in the spleen was made. Medical management was initiated with oral 200 mg albendazole twice a day. Repeated US (every four weeks) was advised. Splenectomy was kept as an end-stage resort if medical treatment failed to reduce the size. The patient was discharged after the fever subsided and was on regular follow-up. Size of lesions in the spleen reduced on progressive US and finally disappeared. Consent was obtained from parents for reporting the case.

## Discussion

Hydatidosis, the cystic echinococcal zoonoses, was first reported by Goeze in 1782. It is one of the more prevalent parasitic infestations in Central Asia including areas of Afghanistan, Iran, Pakistan, Mongolia, Kazakhstan, Uzbekistan and Western China [2]. It is commonly seen in population living in close proximity to animals especially livestock. Rural lifestyle supports the transmission of disease in Pakistan. Due to increased vulnerability and self-neglect, children and young females are particularly prone to develop cystic echinococcosis (CE). A retrospective study in Pakistan, revealed 198 cases of cystic echinococcosis with the highest incidence in 21-30 years age group in three hospitals of the north-eastern region [3]. The location of hydatid cyst is mostly hepatic or pulmonary. Spleen is a relatively less common site for echinococcus. It is usually secondary to systemic dissemination or intraperitoneal spread from a ruptured hepatic cyst. Treatment and diagnosis of splenic cysts is troublesome for many physicians and surgeons.

Children with splenic hydatid cyst usually present with non-specific complaints or it may be detected incidentally. The signs and symptoms are related to splenomegaly, abdominal distension, and compression of adjacent structures [4]. However, it can present with local or referred pain which is postural in majority cases. Some patients may present with complications such as infection of the cyst, intraperitoneal rupture of cyst and fistulization into hollow viscera especially colon followed by upper or lower gastrointestinal bleeding [5]. Although our patient presented with continuous mild left abdominal pain, there were some atypical complaints of high-grade fever and burning micturition associated with non-productive cough and vomiting. On examination, abdominal tenderness in left hypochondrium was found. However, the normal abdominal contour without any visceromegaly or palpable lymph nodes was a non-specific finding in this case.

Diagnosis of splenic hydatid cyst is made by complete blood profile showing eosinophilia, radiological modalities like US and CT scan and specific serum immune-electrophoresis tests [4]. Due to unrelated findings in our case, a few differential diagnoses were kept in mind. As TB is highly prevalent infectious disease in children aged ≤ 14 years in Pakistan, the patient was investigated for abdominal TB [6]. Since the blood culture reports, Montoux test and Genexpert were negative, abdominal TB was excluded. Furthermore, as our patient presented with fever associated with anorexia, vomiting and burning micturition, he was highly suspicious to be diagnosed as a case of pyelonephritis [7]. Although TLC, ESR, and CRP were raised, urinalysis was found to be normal with no cast or culture growth. Additionally, there was no prominent renal finding on X-ray abdomen KUB, abdominal US and CT scan. Clinical signs and symptoms suggested that our patient might have developed abdominal malignancy [8]. However, the abdominal US and CT scan did not show any finding indicative of abdominal malignancy despite lymphadenopathy. Hypodense lesions in the spleen suggested that the patient was not suffering from any abdominal tumor.

As our patient presented with atypical signs and symptoms, insignificant abdominal findings, anemia and leucocytosis on blood profile and was negative for abdominal TB, pyelonephritis, hepatitis, and malignancy, he was further worked up for splenic abscess. The imaging characteristics of splenic hydatid cyst are similar to hepatic or pulmonary cysts. Plain abdominal X-ray findings of splenic hydatid cyst are usually non-specific and may be demonstrated as soft tissue shadow with or without eggshell calcification in left hypochondrium. On abdominal US, splenic hydatid cyst appears as an anechoic round cystic lesion with hyperechoic peripheral calcification. Daughter cysts with detached floating membrane and associated calcification can be seen on an abdominal CT scan [9,10]. Similarly, in our case, abdominal US depicted multiple well defined cystic hypoechoic lesions in the spleen and multiple splenic hypodense lesions associated with enlarged abdominal lymph nodes were noticed on abdominal CT. With no improvement from triple antibiotic therapy, the patient was advised for a serologic test. Abdominal radiographic findings in combination with positive immune-electrophoresis test have a sensitivity of more than 90% for the diagnosis of hydatid disease [4]. The diagnosis of splenic hydatid cyst was confirmed in this patient by positive radiologic findings and IgG antibodies against echinococcus.

The patient was started on albendazole and regular abdominal US on follow-ups were advised. Although anti-helminthic regimen, in combination with surgery, is not considered as the treatment of choice due to poor absorption, low oral bioavailability, and inadequate concentration in cyst cavity to kill parasites, our patient responded well to the treatment [11,12]. The standard treatment of hydatid splenic cysts is splenectomy as it has low morbidity and mortality rate and complete resection removes all parasitic and pericystic tissues, but it was not performed as the fever subsided and US findings improved with medical management [4,13].

## Conclusions

This case report emphasizes that although rare, hydatid splenic cyst should be considered in the differential diagnosis of all cystic masses of spleen especially in children. It may present with vague symptoms that may make it difficult to diagnose clinically. Furthermore, CE being more prevalent in inhabitants of rural areas compared to urban can provide a clue to diagnosis along with specific and baseline investigations that can help in early and better management of such cases.
